# Identification of Immune-Related LncRNA Pairs for Predicting Prognosis and Immunotherapeutic Response in Head and Neck Squamous Cell Carcinoma

**DOI:** 10.3389/fimmu.2021.658631

**Published:** 2021-04-29

**Authors:** Xueying Wang, Kui Cao, Erliang Guo, Xionghui Mao, Lunhua Guo, Cong Zhang, Junnan Guo, Gang Wang, Xianguang Yang, Ji Sun, Susheng Miao

**Affiliations:** ^1^ Department of Head and Neck Surgery, Harbin Medical University Cancer Hospital, Harbin, China; ^2^ Department of Laboratory, Harbin Medical University Cancer Hospital, Harbin, China; ^3^ Department of Surgery, The 2nd Affiliated Hospital of Harbin Medical University, Harbin, China; ^4^ Department of Colorectal Surgery, Harbin Medical University Cancer Hospital, Harbin, China; ^5^ Department of Head and Neck Radiotherapy, Harbin Medical University Cancer Hospital, Harbin, China

**Keywords:** immune-related lncRNA pairs (IRLPs), head and neck squamous cell carcinoma (HNSCC), immune checkpoint inhibitors (ICIs), prognosis, immunotherapies

## Abstract

Long noncoding RNAs (lncRNAs) have multiple functions with regard to the cancer immunity response and the tumor microenvironment. The prognosis of head and neck squamous cell carcinoma (HNSCC) is still poor currently, and it may be effective to predict the clinical outcome and immunotherapeutic response of HNSCC by immunogenic analysis. Therefore, by using univariate COX analysis and Lasso Cox regression, we identified a signature consisting of 21 immune-related lncRNA pairs (IRLPs) that predicted clinical outcome and Immunotherapeutic response in HNSCC. Specifically, it was associated with immune cell infiltration (i.e., T cells CD4 memory resting, CD8 T cells, macrophages M0, M2, and NK cells), and more importantly this signature was strongly related with immune checkpoint inhibitors (ICIs) [such as PDCD1 (r = -0.35, P < 0.001), CTLA4 (r = -0.26, P < 0.001), LAG3 (r = -0.22, P < 0.001) and HAVCR2 (r = -0.2, P < 0.001)] and immunotherapy-related biomarkers (MMR and HLA). The present study highlighted the value of the 21 IRLPs signature as a predictor of prognosis and immunotherapeutic response in HNSCC.

## Introduction

HNSCC is the sixth most common malignant tumor. About 600,000 people worldwide suffer from this disease, and about 300,000 patients die from the disease every year (1). Long-term repeated inflammation is considered to be one of the main causes of the disease, including smoking, drinking, repeated trauma, and human papillomavirus infection (2). HNSCC is characterized by local invasion, regional lymph nodes, and poor prognosis (3). Particularly, patients with advanced HNSCC may require multiple modes of combined treatment, but the quality of the prognosis is not optimistic. Therefore, early detection and in-depth understanding of the characteristics of cancer cells, and accurate diagnosis are the keys to successful treatment. There is an urgent need to study new and sensitive HNSCC tumor prognostic markers to reduce the number of HNSCC patients who are not diagnosed before the onset of the invasive disease.

Cancer immunotherapy aims to enhance the activity of the immune system against cancer and has been the main driving force for personalized treatment (4, 5). In recent decades, immunotherapy has developed rapidly and has become a treatment method for many cancers (6). Several immunotherapies, including immune checkpoint inhibitors, have been developed. In some studies, the expression of PD-L1 in HNSCC is usually higher, with a positive rate of 46% to 100% (7). The reversal of immune rejection mediated by tadalafil and antitumor vaccines also resulted in the up-regulation of PD-L1 in recurrent HNSCC, indicating that immune checkpoint therapy may be equally effective in patients with recurrent HNSCC (8). Relevant research on HNSCC patients is also in full swing, which is expected to improve the survival of HNSCC patients (9, 10). Although these findings support the importance of HNSCC immunology, its molecular mechanism is still unclear, especially for immune-related gnomic effects.

LncRNA is a type of noncoding RNA with 200 nucleotides that does not code for protein (11). lncRNAs are ubiquitous in the genome. They regulate 70% of human gene expression and cannot function in a universal way because they can interact with DNA, RNA, and proteins and exhibit either enhancement or inhibition. Its expression disorder is closely related to the occurrence and development of HNSCC (12, 13). Recent evidence shows that lncRNAs change not only the genome or transcriptome topology but also the immune microenvironment, which contributes to the main phenotype of cancer (14). LncRNA is involved in directing the expression of genes related to immune cell activation, which leads to the tumor’s immune cell infiltration (15). With the development of high-throughput gene sequencing technology and the establishment of large-scale gene expression data sets, cancer researchers are able to accurately identify tumor-related prognostic biomarkers (16). However, there was a batch effect on the detected gene expression levels due to the different platforms and time of testing for gene expression, which may lead to the inaccuracy of the analysis results and bring some difficulties to the comprehensive utilization of data (17). Recently, researchers have provided a new way to solve this difficulty, which can overcome the batch effect of different platforms. The way is to normalize and scale the expression matrix based on the relative ranking of gene expression levels (18, 19). Specifically, we used these immune-related lncRNA expression levels in each sample to compare pairwise and construct IRLPs. In a specific sample, if the expression value of the first irlncRNA is greater than the second irlncRNA, the score of this IRLP in the sample is 1; otherwise, it is 0. The score of each IRLP in all samples was calculated, and IRLPs with low variation were removed (IRLP with a score of 1 or 0 in more than 80% of the sample in any data set) (20). Finally, IRLPs with higher variability were identified for further analysis. This method has produced reliable results in multiple studies. Li et al. validated individualized prognostic markers for pancreatic cancer by integrating IRGPs, presenting a conceivable method for deciding on a preoperative treatment (21). Li et al. constructed IRLPs to predict overall survival in patients with osteosarcoma and to provide potential guidance for patients who might benefit from immunotherapy (22). These studies about IRGPs have important clinical significance for the personalized treatment and prognosis of cancer patients. This method has produced reliable results in multiple studies. Li et al. validated individualized prognostic markers for pancreatic cancer by integrating IRGPs, presenting a conceivable method for deciding on a preoperative treatment (21). Li et al. constructed IRLPs to predict overall survival in patients with osteosarcoma and to provide potential guidance for patients who might benefit from immunotherapy (22). These studies about IRGPs have important clinical significance for the personalized treatment and prognosis of cancer patients.

However, there have been no studies on the clinical relevance and prognostic significance of IRLPs in HNSCC.

In conclusion, in terms of the accuracy of cancer prediction models, the combination of two biomarkers is better than simple genes (19). We integrated the sequencing samples of 546 HNSCC patients based on the TCGA data set. Univariate COX analysis and Lasso Cox regression are used to determine reliable IRLPs. These IRLPs signatures can predict the clinical outcome of HNSCC and establish a prognostic model of risk associated with immune gene pairs. We found that IRLPs are powerful prognostic biomarkers and predictors of HNSCC.

## Materials and Methods

### Clinical Sample and Data Collection

Gene expression quantification data (FPKM and counts format) for HNSCC were downloaded from TCGA (https://portal.gdc.cancer.gov/). Then 44 normal samples and 502 HNSCC samples were obtained. The RNA expression matrix was extracted separately by annotations using the Gencode (GENCODE v 26) GTF file and normalized. Genes whose expression was “0” in 90% of HNSCC patients were removed. Clinical data were downloaded from the UCSC Xena website (https://xena.ucsc.edu/). To analyze the correlation of lncRNA expression signatures with the prognosis of HNSCC patients, we filtered out samples without survival information. Then, we selected a total of 499 patients. Significant lncRNA-pathway pairs across 33 cancer types with each lncRNA having an activity in immune pathways (lncRES) score> 0.995 and a false discovery rate (FDR) < 0.05 were downloaded from Immlnc (http://biobigdata.hrbmu.edu.cn/ImmLnc/index.jsp) (23). The list of immune-related lncRNAs in HNSCC was extracted separately. Stromal scores and immune scores of HNSCC were calculated by applying the ESTIMATE algorithm and downloaded from the website (https://bioinformatics.mdanderson.org/estimate/index.html) (24).

### Analysis of Differentially Expressed lncRNAs

We obtained DElncRNAs between normal and tumor tissues, where P value < 0.05 and log2-fold change (FC) > 1.5 were used as the cutoffs by using the R package ‘edgeR’ (25). Then, we filtered DEirlncRNAs by matching the list of immune-related lncRNA in HNSCC. The R package ‘heatmap’ was used to display the eight selected irlncRNAs.

### Identification of Prognostic-Related IRLPs in Patients With HNSCC

We then used the lncRNA expression levels of these lncRNAs in each sample for pairwise comparison to construct irlncRNAs. In a specific sample, if the expression value of the first irlncRNAs is greater than that of the second irlncRNAs, the score of this IRLPs in the sample is 1; otherwise, it is 0. The score of each IRLP in all samples was calculated, and IRLPs with low variation were removed (IRLPs with a score of 1 or 0 in less than 20% of the sample in any data set). Finally, IRLPs with higher variability were identified for further analysis. Univariate Cox regression analysis was performed on these IRLPs in the TCGA cohort and IRLPs with p < 0.0001 were considered prognostic-related IRLPs and used for subsequent analysis.

### Construction and Evaluation of Signatures Based on IRLPs

Lasso Cox regression analysis was performed on the above-mentioned prognostic-related IRLPs, and finally an optimal model composed of 21 IRLPs was determined. Subsequently, the optimal model based IRLPs signature of each patient was calculated. In the 3-year overall survival TCGA cohort, time-dependent ROC curve analysis was used to determine the optimal cutoff value for IRLPs signature (22, 26). According to the cutoff value of the IRLPs signature, patients were divided into high-risk group and low-risk group. The log-rank test was used to evaluate the overall survival difference between the low-risk group and the high-risk group, and the KM survival curve was drawn. ROC curve analysis was used to evaluate the sensitivity and specificity of IRLPs. An ROC curve, including clinical characteristics, was drawn, and the AUC was calculated. Finally, univariate, and multivariate Cox regression analyses were used to investigate whether the prognostic value of the IRLPs was affected by other clinical characteristics.

### Construction and Evaluation of Nomograms

We combined the clinical characteristics of the TCGA data set with the IRLPs signature to construct a nomogram. We used the C index to evaluate the discriminative power of the nomogram and drew a calibration chart to evaluate the accuracy of the nomogram. We then compared the decision curve analysis between the clinical characteristics model and the combined model, including gene signature.

### Estimation of Immune Infiltration

Estimation of STromal and Immune cells in MAlignant Tumor tissues using Expression data (ESTIMATE) is a tool for predicting tumor purity and the presence of infiltrating stromal/immune cells in tumor tissues using gene expression data. ESTIMATE algorithm is based on single sample Gene Set Enrichment Analysis and generates three scores.

First, the immune infiltration assessment was performed using the “microenvironment cell population count (MCP-counter)” method (27). Using the normalized FPKM expression matrix converted by log2 as input, the absolute abundance scores of 8 immune cells and 2 stromal cells populations are generated through the “MCP-counter” package. Research shows that immune cell infiltration assessed by the MCP-counter algorithm performs well when comparing between samples (28). Subsequently, CIBERSORT was used to infer the relative proportion of 22 infiltrating immune cells in each sample for supplementation.

### Analysis of the Immunosuppressive Molecules Expressing Related to ICIs

To study the relationship between the model and the expression level of genes related to ICIs, we performed ggstatsplot package and violin plot visualization.

### Gene Set Enrichment Analysis

GSEA software (version 4.0.1) was used to perform gene set enrichment analysis between high-risk and low-risk groups. Recognized the enriched terms in IMMUNE and KEGG in high-risk group and low-risk group respectively. P < 0.05 and False discovery rate (FDR) <0.05 are considered statistically significant.

### Statistical Analysis

Except for gene set enrichment analysis, all statistical analyses involved in this research were conducted using the R software (version 4.0.3, R Foundation for Statistical Computing, Vienna, Austria). Unless otherwise stated, p < 0.05 is considered statistically significant.

## Result

### Construction and Evaluation of IRLPs Signature

**Figure 1:**
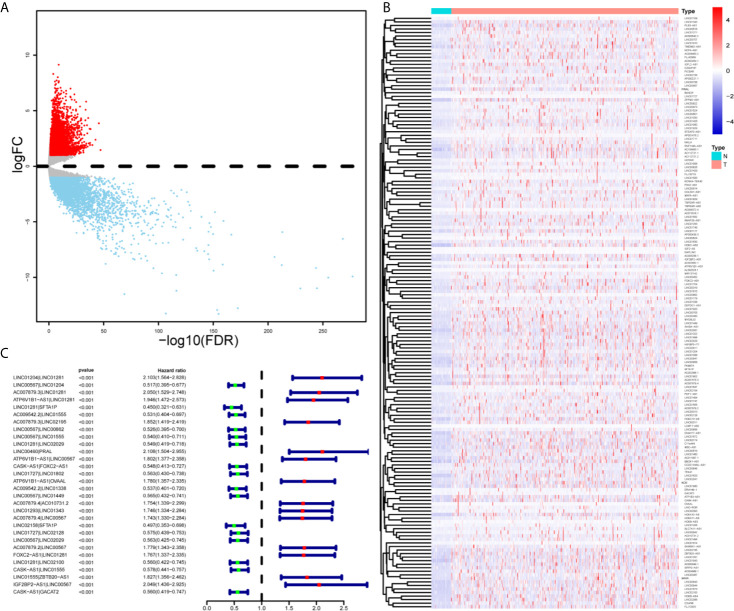
Identification of DEirlncRNAs and IRLPs using TCGA datasets. **(A)** For the volcanic map of 6720 differentially expressed genes, red points represent log2FC >1.5; blue points represent log2FC<-1.5, FDR < 0.05. **(B)** Heat maps of 167 immune related differential lncRNAs in normal and tumor samples. **(C)** Forest map showing 30 DEirlncRNA pairs related to prognosis identified by univariate COX analysis.

**Figure 2:**
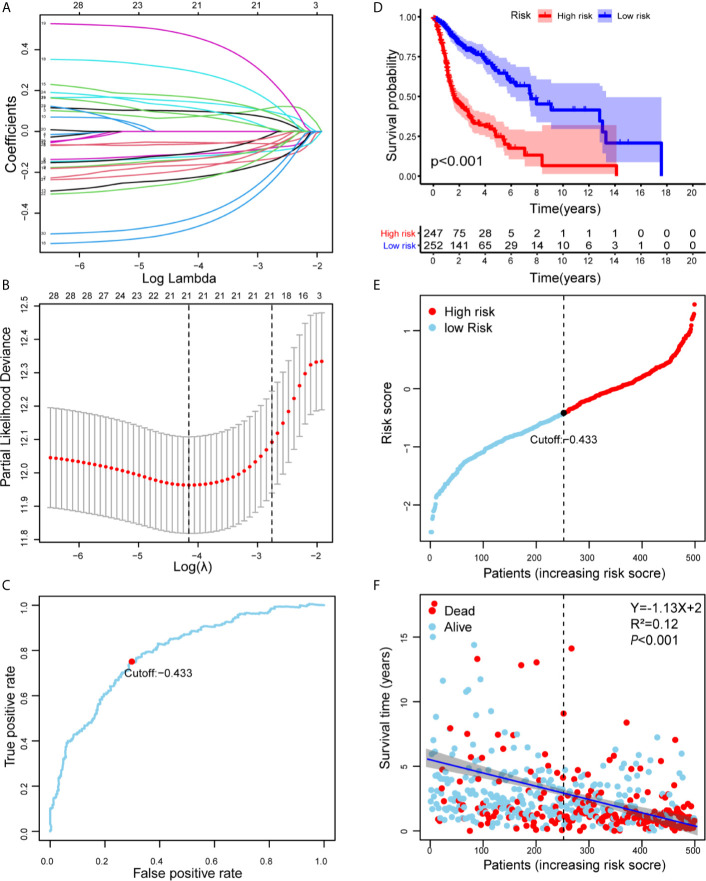
Risk assessment model for prognosis prediction. **(A)** Validation was performed for tuning parameter selection through the Lasso regression model for OS. **(B)** Elucidation for LASSO coefficient profiles of prognostic IRLPs. **(C)** Time-dependent ROC curve of the IRLPs signature in the TCGA cohort. The optimal cutoff value of the IRLPs signature is -0.433; patients are divided into the high-risk group and the low-risk group according to the cutoff value. **(D)** Patients were sorted by increasing risk score in the HNSCC set. **(E)** The Kaplan-Meier survival curve with log-rank test was drawn to demonstrate the relationship between risk model and OS. Compared with the high-risk group, patients in the low-risk group experienced a longer survival time. **(F)** The survival time and survival status of patients with HNSCC worsened as the risk score increased (Y=-1.13X+2, R^2^=0.12).

As shown in **Supplementary Figure 1**, we first retrieved the transcriptome analysis data of HNSCC from the TCGA database. Next, we annotated the data with GTF files, and we applied the edgR package for difference analysis, based on the normal and tumor samples in TCGA, A total of 6720 differentially expressed genes was screened, of which 4063 were upregulated and 2657 were down-regulated (**Figure 1A**). (|log2FC|>1.5, FDR <0.05). Further, the lack of clinical information and duplicate samples were removed, and a total of 499 cases were included in survival-related analysis. We crossed the lncRNAs (2391) related to HNSCC immunity obtained by Immlnc with the lncRNAs highly expressed in HNSCC to obtain 167 immunologically related differential lncRNAs (Because the expression value of lncRNAs in tumor samples is very low and cannot provide valuable reference in subsequent experiments, we chose the highly expressed lncRNAs for the study.) The differential expression of 167 lncRNAs was visualized in **Figure 1B**. Then paired analysis of these lncRNAs, a total of 7719 valid differential expression IRLPs were identified. These gene pairs were subjected to univariate COX analysis (p < 0.0001). Finally, 30 IRLPs related to prognosis were screened out (**Figure 1C**). To prevent overfitting, these prognostic lncRNA pairs were subjected to Lasso Cox regression analysis, and 21 IRLPs were obtained (**Figures 2A, B**). The 21 IRLPs (**Table 1**) were selected to construct the signature. (The list of lncRNA pairs, immune pathways and coefficients are shown in **Table 1**). We use the time-dependent receiver operating characteristic (ROC) curve to determine the cutoff value for the best IRLPs signature. The optimal cutoff value for IRLPs signature is -0.433 (**Figure 2C**). According to the cutoff value, patients were divided into the high-risk group and the low-risk group. We find that compared with patients in the low-risk group, patients’ overall survival rate in the high-risk group was significantly lower (**Figure 2D**). With the increase of the risk score, the patient’s survival time shortened gradually, and the mortality rate gradually increased (**Figures 2E, F**).

**Table 1:** Information of 21 IRLPs.

IRLPs	LncRNA Pair1	Immune pathway*	LncRNA Pair2	Immune pathway*	Coefficient
LINC00567|LINC01204	LINC00567	Cytokine Receptors	LINC01204	Cytokines	-0.16528
AC007879.3|LINC01281	AC007879.3	Antigen Processing and Presentation	LINC01281	Natural Killer Cell Cytotoxicity	0.071247
LINC01281|SFTA1P	LINC01281	Natural Killer Cell Cytotoxicity	SFTA1P	Natural Killer Cell Cytotoxicity	-0.12353
AC009542.2|LINC01555	AC009542.2	Cytokines	LINC01555	Antigen Processing and Presentation	-0.11187
AC007879.3|LINC02195	AC007879.3	Antigen Processing and Presentation	LINC02195	Natural Killer Cell Cytotoxicity	0.099465
LINC00567|LINC00862	LINC00567	Cytokine Receptors	LINC00862	Cytokines	-0.05372
LINC00567|LINC01555	LINC00567	Cytokine Receptors	LINC01555	Antigen Processing and Presentation	-0.13669
LINC00460|PRAL	LINC00460	Cytokines	PRAL	Cytokine Receptors	0.145737
CASK-AS1|FOXC2-AS1	CASK-AS1	Antigen Processing and Presentation	FOXC2-AS1	Cytokines	-0.22806
LINC01727|LINC01802	LINC01727	Chemokine Receptors	LINC01802	Cytokines	-0.13905
ATP6V1B1-AS1|OVAAL	ATP6V1B1-AS1	Cytokines	OVAAL	Cytokines	0.167943
AC009542.2|LINC01338	AC009542.2	Cytokines	LINC01338	Cytokines	-0.47794
AC007879.4|AC010731.2	AC007879.4	Cytokines	AC010731.2	Antigen Processing and Presentation	0.297284
LINC01293|LINC01343	LINC01293	Cytokines	LINC01343	Interleukins Receptor	0.469817
LINC02158|SFTA1P	LINC02158	Antimicrobials	SFTA1P	Natural Killer Cell Cytotoxicity	-0.18683
LINC01727|LINC02128	LINC01727	Cytokine Receptors	LINC02128	Antimicrobials	-0.2438
AC007879.2|LINC00567	AC007879.2	Cytokines	LINC00567	Cytokine Receptors	0.117046
LINC01281|LINC02100	LINC01281	Cytokine Receptors	LINC02100	Antimicrobials	-0.10826
CASK-AS1|LINC01555	CASK-AS1	Antigen Processing and Presentation	LINC01555	Antigen Processing and Presentation	-0.06256
IGF2BP2AS1|LINC00567	IGF2BP2-AS1	Cytokines	LINC00567	Cytokine Receptors	0.081704
CASK-AS1|GACAT2	CASK-AS1	Antigen Processing and Presentation	GACAT2	Cytokines	-0.42858

*Immune pathway was annotated by website Immlnc (http://biobigdata.hrbmu.edu.cn-/ImmLnc/index.jsp).

### Correlation Between IRLPs and Clinical Characteristics

**Figure 3:**
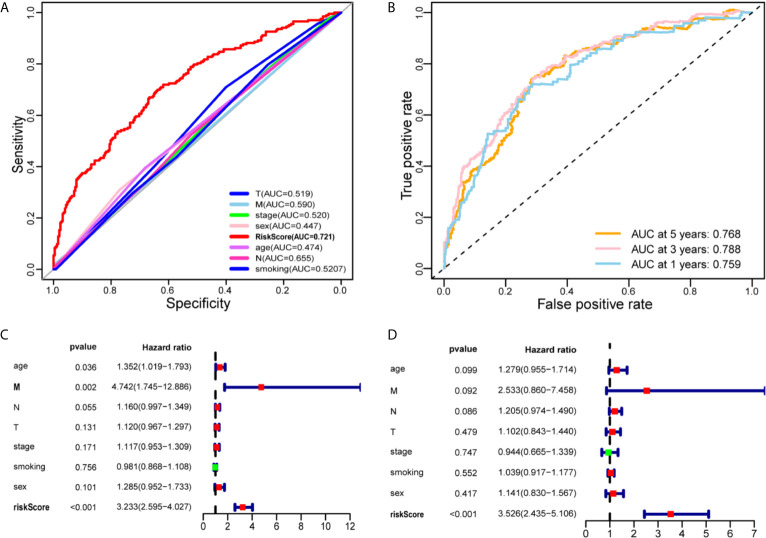
Evaluate the predictive ability of the IRLPs signature. **(A)** A comparison of ROC curves with other common clinical characteristics showed the superiority of the IRLPs signature. **(B)** The 1-, 3-, and 5-year ROC curve of the optimal model suggested that all AUC values were over 0.75. **(C)** Forest plot of univariate Cox regression results shows that risk score (P < 0.001) and Metastasis (P < 0.05) are prognostic related factors. **(D)** Forest plot of multivariate Cox regression results shows that risk score (P < 0.001) is an independent influencing factor for prognosis.

**Figure 4:**
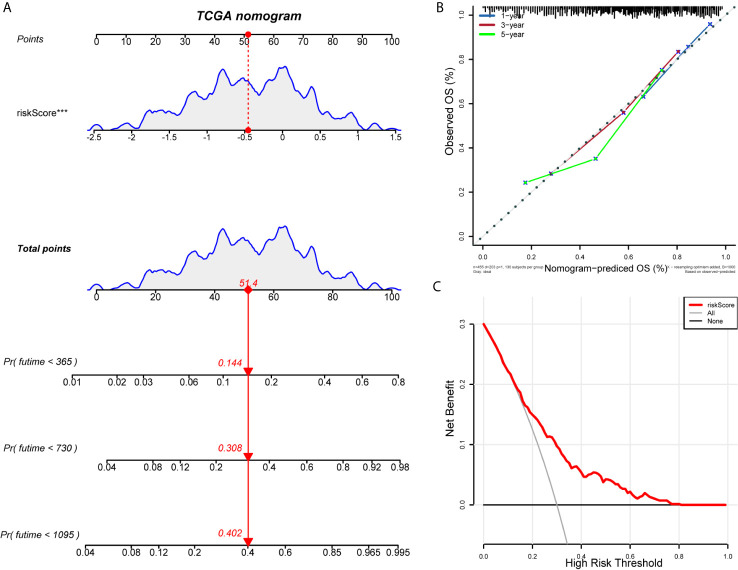
Establishment and evaluation of nomogram model. **(A)** Nomogram predicting the probability of TCGA patient’s mortality based on IRLPs and clinical variables. **(B)** Calibration curves of the nomogram for 1, 3 and 5 years. **(C)** Decision curve analysis of the nomograms based on the IRLPs signature.

We construct the ROC curve of the IRLPs signature, TNM stage, age, sex, and smoking. The area under the curve (AUC) of the IRLPs signature is 0.721 (**Figure 3A**), which shows that our signature has excellent predictive power. Then we draw the time-dependent ROC curve of the IRLPs signature. We find that the area under the IRLPs signature curve respectively: 1 year: 0.759; 3 years: 0.788; 5 years: 0.777 (**Figure 3B**). This shows that our model has a good predictive ability for patients with 5-year survival, 3-year survival, and 1-year survival. Next, we assessed the prognostic value of the HNSCC risk score. In the univariate analysis, we find that the risk score was significantly correlated with the overall survival (OS) (HR = 3.233, 95% CI = 2.233 - 4.027, P < 0.001). Multivariate analysis shows that the risk score is an effective independent prognostic predictor of OS (HR = 3.526, 95% CI = 2.435 - 5.106, P < 0.001) (**Figures 3C, D**). In order to further improve the accuracy of the prediction, we constructed a new nomogram based on the IRLPs signature (**Figure 4A**). The nomogram C-index is 0.729. By calculating the total score, oncologists can easily obtain the probability of OS predicted by the nomogram of a single patient. We also use the calibration curve to evaluate the model’s prediction accuracy (**Figure 4B**). The results show that the prediction calibration curve of the three calibration points in 1, 3, and 5 years is close to the standard curve, which indicates that the model has good predictive performance. In addition, we also use the DCA (decision curve) to evaluate the reliability of the model (**Figure 4C**). It can be seen that the profit of this model is significantly higher than the limit curve, so it has good reliability.

### The Relationship Between IRLPs Signature and Immune Cell Infiltration

**Figure 5:**
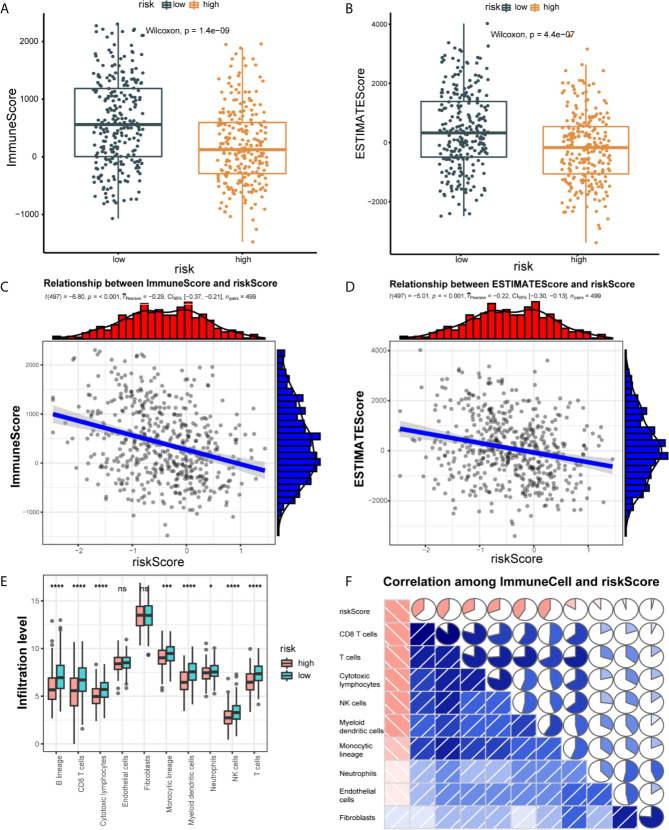
Correlation of 21 IRLPs features with immune cell infiltration and immune scores. **(A)** The Wilcoxon rank-sum test was used to compare differences in immune scores between low- and high-risk groups. (P < 0.001) **(B)** The Wilcoxon rank-sum test was used to compare differences in ESTIMATE scores between low- and high-risk groups. (P < 0.001) **(C)** Spearman’s correlation coefficients were computed to investigate the potential relationship between risk score and immune scores. **(D)** Spearman’s correlation coefficients were computed to investigate the potential relationship between risk score and ESTIMATE scores. **(E)** The Wilcoxon rank-sum test compared the absolute abundance scores of 8 immune cells and 2 stromal cells populations in two groups of patients. **(F)** Spearman’s correlation coefficients were computed to investigate the potential relationship between absolute abundance scores of immune cells and stromal cells and risk score. The area of fan represents the degree of correlation (Red represents a negative correlation and blue represents a positive correlation). ns, no significance.

We explore the difference in immune cell infiltration between the two groups. Based on the ESTIMATE algorithm, we first calculate the Immune score and ESTIMATE score of each HNSCC sample. As shown in **Figures 5A, B**, compared with the low-risk group, the Immune score (190.71 *vs* 608.83, p < 0.001) and ESTIMATE score (-213.51 *vs* 402.27, p < 0.001) of the high-risk group are lower and negatively correlated with the risk score (correlation coefficients are -0.22 and -0.29, p < 0.001) (**Figures 5C, D**). Next, we used the MCP-counter method to calculate the abundance of 8 immune cells and 2 stromal cells. Significant differences were observed between the two groups of patients. Compared with patients in the high-risk group, the eight cell populations in the low-risk group are more abundant (B cell lineage, CD8(+) T cells, cytotoxic lymphocytes, monocyte lineage cells, myeloid dendritic cells, medium Sex granulocytes, NK cells, T cells) (**Figure 5E**).

**Figure 6:**
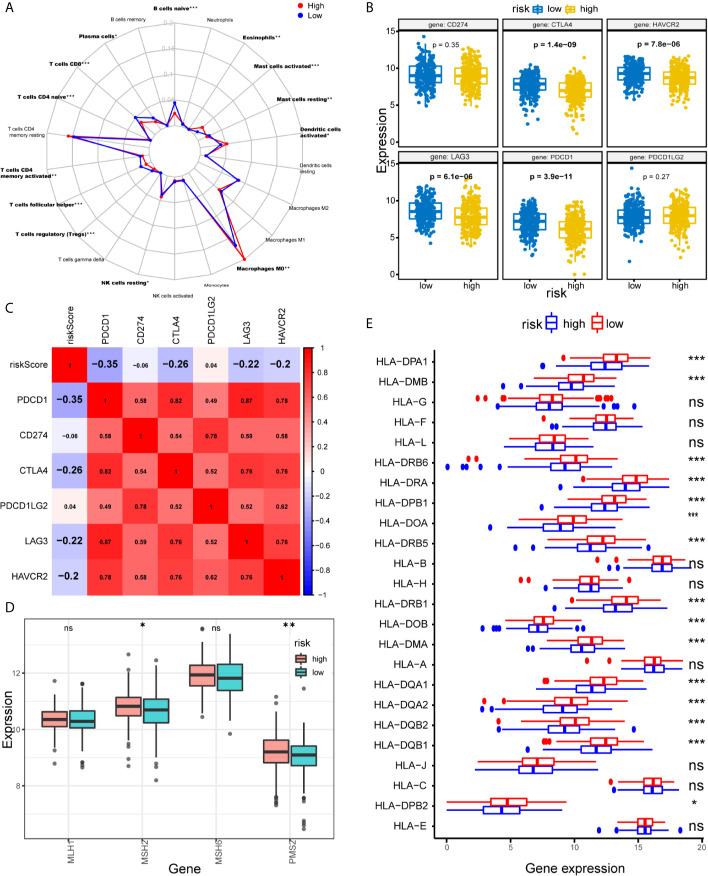
Assessments of the relationship between immune cells, HLA, MMR genes, ICIs, and IRLPs signature. **(A)** Radar chart of the relationship between 22 immune cell infiltration and IRLPs signature grouping. (Wilcoxon test). **(B)** The different expressions of ICIs among risk groups as defined by the 21 IRLPs signature. Results revealed that CTLA4, HAVCR2, LAG3 and PDCD1 were overexpressed in low-risk group (all P<0.001). **(C)** Spearman’s correlation coefficients were computed to investigate the potential relationship between our IRLPs signature and ICIs. The highlighted ones represent P<0.001. **(D)** The different expressions of MMR genes among risk groups as defined by the 21 IRLPs signature. (Wilcoxon test). **(E)** The box plot showed that most of the HLA gene families were highly expressed in the low-risk group. (Wilcoxon test). *P < 0.05, **P < 0.01, ***P < 0.001. ns, no significance.

We further explored the relationship between the immune cell infiltration and the risk score. The result show that the degree of immune cell infiltration is negatively correlated with the risk score (**Figure 5F**). Subsequently, we used CIBERSORT to further supplement the relative proportion of 22 immune infiltrating cells in each sample (**Figure 6A**). The relative proportions of B cells naive, mast cells resting, plasma cells, T cells CD4 memory activated, T cells CD8, T cells follicular helper, and T cells regulatory (Tregs) in the low-risk group are higher. The relative proportions of dendritic cells activated, eosinophils, T cells CD4 naive, macrophages M0, mast cells activated, and NK cells resting were relatively high in the high-risk group. This indicates that there are great differences in immune cell infiltration between high- and low-risk groups. It is worth noting that the radar chart shows that T cells CD4 memory resting and M0 macrophage infiltration rate are higher in all patients.

### The Relationship Between IRLPs Signature and Immune Checkpoint

Tumor immunotherapy using ICIs has become a promising treatment for advanced HNSCC (29). To further study the relationship between IRLPs and immunity, we explored the risk score and ICIs-related biomarkers correlation. The results showed that in the low-risk group, the expression levels of PDCD1, CTLA4, LAG3, and HAVCR2 were upregulated (all P < 0.001), and the risk score was negatively correlated to PDCD1 (r = -0.35, P < 0.001), CTLA4 (r = -0.26, P < 0.001), LAG3 (r = -0.22, P < 0.001) and HAVCR2 (r = -0.2, P < 0.001), indicating that the low-risk group had benefited more from immunotherapy (**Figures 6B, C**).

### The Relationship Between Risk Score, MMR Gene and HLA Gene Family

Solid tumors lacking the mismatch repair (MMR) genes are usually immunogenic and exhibit extensive infiltrating T cells, making them highly sensitive to ICIs (30). We evaluated the correlation between IRLPs signals and four key MMR genes (MSH6, MLH1, PMS2, MSH2). The expression levels of PMS2 and MSH2 in the high-risk group were upregulated (PMS2 and MSH2 were p < 0.05 and p < 0.05, respectively), suggesting that high-risk group did not benefit from immunotherapy as much (**Figure 6D**). Furthermore, immune escape is a hallmark of cancer, and the ability to present new antigens through the loss of human leukocyte antigen (HLA) may help immune escape (31). We find that HLA family plays a certain role in the sensitivity difference of immunotherapy, as shown in **Figure 6E**, HLA family was downregulated in the high-risk group, which led to tumor immune evasion, and may be related to immunotherapy insensitivity.

### Gene Set Enrichment Analysis

**Figure 7:**
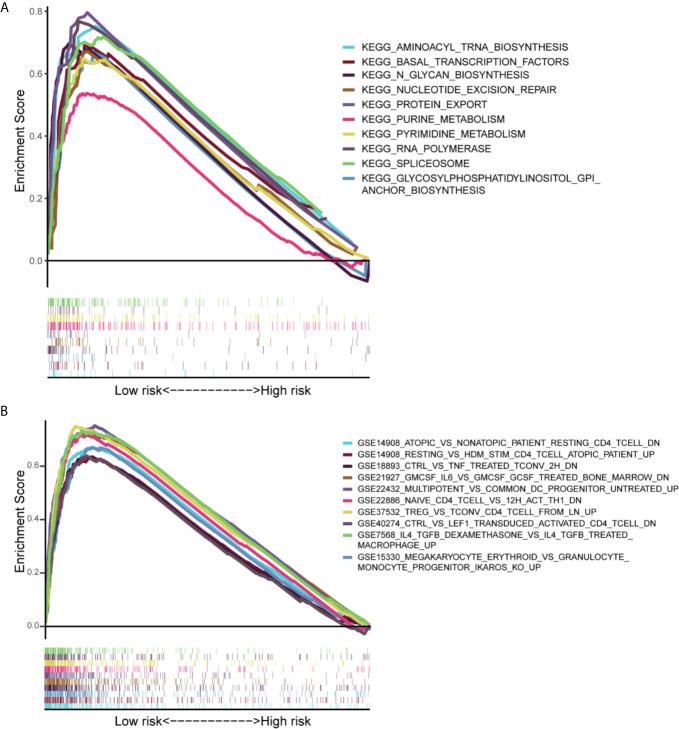
Results of gene set enrichment analysis in the TCGA cohort. **(A)** The significantly enriched KEGG subset of canonical pathways by GSEA. **(B)** The significantly enriched Immunologic gene sets by GSEA.

Gene set enrichment analysis (GSEA) was performed to determine the gene sets enriched in different IRLPs subgroups. The gene sets of the IRLPs-low samples were enriched in nucleotide excision repair and CD4 T cell, TNF, IL6, etc. (**Figure 7**).

## Discussion

HNSCC is a solid malignant tumor with strong immunogenicity, its incidence increasing rapidly worldwide. Advances in surgical techniques and comprehensive treatment techniques have improved the local control rate and quality of life of HNSCC patients. Still, in recent decades, the survival rate has not increased significantly. In addition, the 5-year survival rate of patients with this disease is only 40% - 50% (32). Platinum-based chemotherapy, combined with cetuximab, is the standard treatment for relapsed or metastatic HNSCC. However, there are problems such as easy relapse and short median survival after treatment (33–35). ICIs based on PD-1/PDL-1 monoclonal antibodies have become a new clinical treatment option for advanced HNSCC. Both pembrolizumab and nivolumab have been approved by the FDA for relapsed or metastatic HNSCC that have failed platinum-based therapy (36). Important studies based on prognostic signals of immune gene expression have shown that gene expression scores can predict the risk of recurrence and the effect of immunotherapy (37, 38).

Given that the results of single antibody drugs are limited, and there are many connections between the occurrence and development of HNSCC and the immune microenvironment, the strategy of multiple immunotherapies may have better prospects (39). Therefore, it is necessary to use IRLPs to establish prognostic indicators. Reliable prognostic biomarkers can identify patients with poor prognosis and inform patients who may benefit from other systemic treatments. Hence, they have more direct clinical significance.

In our study, based on the HNSCC immune-related lncRNAs data set, IRLPs that significantly affect the OS of patients were constructed. These IRLPs can help identify candidate immune-related biomarkers or therapeutic targets. Unlike traditional prognostic models, the pairwise comparison and score calculation of each IRLPs are based entirely on the lncRNA expression of the same patient, so our IRLPs signature does not have to be standardized on the gene expression profile sequencing platform from different patients. Previous research has proved the effectiveness of this method (40). Therefore, the prognostic signature can overcome the batch effect of different platforms and does not require data scaling and normalization. This approach has been reported to be robust in other cancer-related studies (20, 41).

First, we retrieved raw data of lncRNAs from TCGA, performed a differential co-expression analysis to classify the differentially expressed irlncRNAs (DEirlncRNAs), and validated lncRNA-pairs using an improved method of cyclically single pairing along with a 0-or-1 matrix. Second, we performed univariate analysis combined with a modified Lasso Cox regression, including procedures of cross-validation, multi-times repeat, and included 21 IRLPs with prognostic significance. Third, we calculated each AUC value of ROC curve to obtain the best model and determined the best cutoff value of the IRLPs signature according to the ROC curve differentiate the high or low risk-group among patients with HNSCC. Fourth, we evaluated this novel model under various clinical settings, including survival, clinicopathological characteristics, tumor-infiltrating immune cells, chemotherapy, and checkpoint related biomarkers. Among the 21 IRLPs, a total of 28 lncRNAs were included. These lncRNAs participate in the occurrence and development of HNSCC. Specifically, CASK-AS1 | GACAT2 may play an important role in the screening and prognosis of HNSCC. The experimental results of Tan et al. showed that the preoperative plasma GACAT2 levels of gastric cancer patients were significantly higher than that after surgery (P=0.031). Therefore, they believed that plasma GACAT2 could be used as a tumor marker for the screening and prognosis prediction of cancer patients (42). The results of Liu et al. identified 5 lncRNAs (TSPEAR-AS, ​​CASK-AS1, MIR137HG, Part1, LSAMP-AS1) as potential prognostic markers and therapeutic targets for laryngeal cancer, which is consistent with our results (43). In addition, the LINC00460 promotes tumor progression through sponge miR-4443 in HNSCC (44). The loss of major histocompatibility complex I (MHC I) molecules is an important mechanism for HNSCC cells to evade immune surveillance. However, LINC02195 is a crucial regulator of MHC I molecules (45). Moreover, novel lncRNA SFTA1P promotes tumor growth by downregulating miR-4766-5p via the PI3K/AKT/mTOR signaling pathway in hepatocellular carcinoma (46). However, studies have shown that IL-22 can induce lncRNA H19 to activate mTOR signal transduction, thereby preventing liver damage. This shows that the mTOR signaling pathway has potential and broad therapeutic prospects (47). Linc01555 promotes proliferation, migration, and invasion of gastric carcinoma cells by interacting with the Notch signaling pathway. LncRNA FOXC2-AS1 enhances FOXC2 mRNA stability to promote colorectal cancer progression via activation of the Ca-FAK signal pathway (48). These studies support that our risk scoring model can be used as an indicator to predict the prognosis of HNSCC patients. In addition, our scoring system has strong predictive power for OS: the AUC values for predicting 1-year, 3-year, and 5-year overall survival rates are 0.759, 0.788, and 0.768, respectively.

Survival analysis and univariate/multivariate Cox proportional hazard analysis proved the prognostic value and that our IRLPs signature is an independent prognostic factor. It is worth noting that the TNM staging, smoking, and age prognostic models did not show good predictive values. Therefore, our risk scoring model may be more helpful for clinicians to predict the survival of HNSCC patients.

In our study, correlation analysis showed that 21 IRLPs signatures were positively correlated with MMR genes MSH2 and PMS2, indicating that these patients had poor responses to ICIs. Immune escape is an important mechanism for the occurrence and development of malignant tumors. Losing the ability to present neoantigens through human leukocyte antigen (HLA) loss may facilitate immune evasion (31). The HLA family plays a certain role in the sensitivity difference of immunotherapy. The results show that the HLA family is downregulated in the high-risk group, which leads to tumor immune evasion, which may be related to immunotherapy insensitivity. Next, we explored the relationship between IRLPs and known predictive biomarkers for immunotherapy. According to the signature characteristics of 21 IRLPs, ICIs, including PDCD1, CTLA4, LAG3, and HAVCR2, were highly expressed in the low-risk group (P<0.001), whose survival rate was higher. When exploring the correlation between the 21 IRLPs values and PDCD1, CTLA4, LAG3, and HAVCR2, the 21 IRLPs signatures were significantly negatively correlated with ICIs expression. There is strong evidence that patients with low-risk scores may benefit more from immunotherapy. Generally, PDCD1+ tumors respond better to anti-PD-1 therapy than PDCD1-tumors (49, 50). However, we found inconsistent results in HNSCC. That is, when evaluating anti-PD-1 therapy in the setting of platinum-refractory relapsed or metastatic HNSCC, CHECKMATE-141 failed to show a significant correlation between PD-L1 expression and tumor response or survival (51). The main reason may be the lack of uniformity in the measurement and the variability used to define the PD-1 positivity threshold. Moreover, we believe that the intensity and location of PD-1 expression detected by immunohistochemistry are more valuable than the PD-1 expression value measured by the transcriptome data. Therefore, further research is needed to clarify the relationship between PD-1 and IRLPs. In summary, our findings reveal the important value of HNSCC immunotherapy.

Understanding the overview of the tumor microenvironment (TME) may help find new ways to treat HNSCC or change TME to improve the effectiveness of immunotherapy. Macrophages that reside within the TME are known as tumor-associated macrophages (TAMs) (52). TAMs and other TME members make up the tumor ecosystem. In most situations, all members of the TME consume oxygen and nutrients from the host for their phenotypic and functional performance (53, 54). Thus, metabolites are accumulated in the TME and recycled from cell to cell. In particular, the metabolites, as messengers for cell-cell contact, which are derived from the TME (tumor cells, T cells, mast cells, cancer-associated fibroblasts, adipocytes, except TAMs), are ingested by TAMs to change their phenotype and function. In turn, TAMs promote tumor progression via metabolic reprogramming, which is triggered by the metabolites that are shuttled in the TME (55). Given the importance of TAMs in tumorigenesis and development, there has been considerable interest in therapeutic strategies that target macrophages, which can be roughly divided into depletion or alteration of TAM protumoral activities (56). Most clinical studies believe that combination therapy is necessary to maximize the benefit of cancer patients (57), these strategies are currently in evaluation either to augment tumor immunity during standard chemotherapy or radiation therapy, or in combination with T cell-directed immunotherapy (58). Therefore, the assessment of the immune status in the tumor microenvironment is necessary for a comprehensive understanding of the real-time status of the tumor.

First, we verified the correlation between the ESTIMATE score, the immune score, and the risk score. Between the two different risk groups, significant differences were observed in the relative fraction of immune cells infiltrating the tumor tissue. The composition of immune cells between the two subgroups of IRLPs was further analyzed. We find that dendritic cells activated, macrophages M0, mast cells activated, and NK cells resting in the high subgroup of IRLPs are more abundant, while B cells naive, mast cells resting, plasma cells, T cells CD4 memory activated, T cells CD8, T cells follicular helper and T cells regulatory (Tregs) are more common. A large number of studies have shown that dense infiltration of T cells, especially T cells CD8, predicts a good prognosis (59–61). In most tumors, M2 macrophages are the main subtype of macrophages and have been shown to be involved in tumor growth and development with chronic inflammation and aggressive phenotypes. These cells are found in breast, bladder, and ovarian cancer. The prognosis is poor in gastric cancer and glioma (62–66). Our research results support these conclusions.

In addition, our results show differences in biological processes and immune infiltration between the two groups. The results of GSEA show that many immune-related pathways are enriched in the low-risk group. From this perspective, patients in the low-risk group are also more likely to benefit from immunotherapy.

It should be admitted that our research still has some limitations. First, conclusions about the efficacy of immunotherapy have not been confirmed in patients with HNSCC, and further research is needed to verify our results. Finally, although the signature we constructed has excellent performance in predicting immunotherapy and prognosis, it is still the tip of the iceberg in the current immunotherapy field, and a lot of research is needed to enrich and improve.

In short, IRLPs is a promising immune-related prognostic biomarker. IRLPs grouping may help distinguish immune and molecular characteristics and predict patient prognosis. IRLPs may be a potential prognostic indicator of immunotherapy. This may open a new chapter in HNSCC immunotherapy.

## Data Availability Statement

The original contributions presented in the study are included in the article/**Supplementary Material**. Further inquiries can be directed to the corresponding authors.

## Ethics Statement

Ethical review and approval was not required for the study on human participants in accordance with the local legislation and institutional requirements. Written informed consent for participation was not required for this study in accordance with the national legislation and the institutional requirements.

## Author Contributions

XW and KW conceptualized the project, all data analysis, and wrote the first draft of the manuscript. EG, XM, LG, CZ, JG, and GW contributed to processing, analysis, and interpretation of the data. JS and SM contributed to guide the data analysis, and manuscript writing. All authors contributed to the article and approved the submitted version.

## Funding

This work was supported by Postdoctoral Scientific Research Developmental Fund of Heilongjiang Province (LBH-Q18088).

## Conflict of Interest

The authors declare that the research was conducted in the absence of any commercial or financial relationships that could be construed as a potential conflict of interest.

## Abbreviations

HNSCC, Head and neck squamous cell carcinoma; OS, Overall survival; IRLPs, Immune related lncRNA pairs; TCGA, The Cancer Genome Atlas; LASSO, Least absolute shrinkage and selection operator; ICIs, Immune checkpoint inhibitors; FPKM, Fragments per Kilobase Million; DElncRNAs, Differentially expressed lncRNAs; CIBERSOFT, Celltype Identification by Estimating Relative Subsets of RNA Transcripts; GSEA, Gene set enrichment analysis; MMR, Mismatch repair; TMB, Tumor mutation burden; ROC, Receiver operating character; AUC, Area under curve; LncRNAs, Long non-coding RNAs; FDR, False discovery rate; MCP-counter, microenvironment cell population count.
