# Supplementary material for: Identification of Immune-Related LncRNA Pairs for Predicting Prognosis and Immunotherapeutic Response in Head and Neck Squamous Cell Carcinoma
Source: Front Immunol. 2021 Apr 29;12:658631. doi: 10.3389/fimmu.2021.658631 (PMC8116744; doi:10.3389/fimmu.2021.658631)
Supplement: Supplementary file 2 [file Image_1.pdf]

Supplementary Figures:

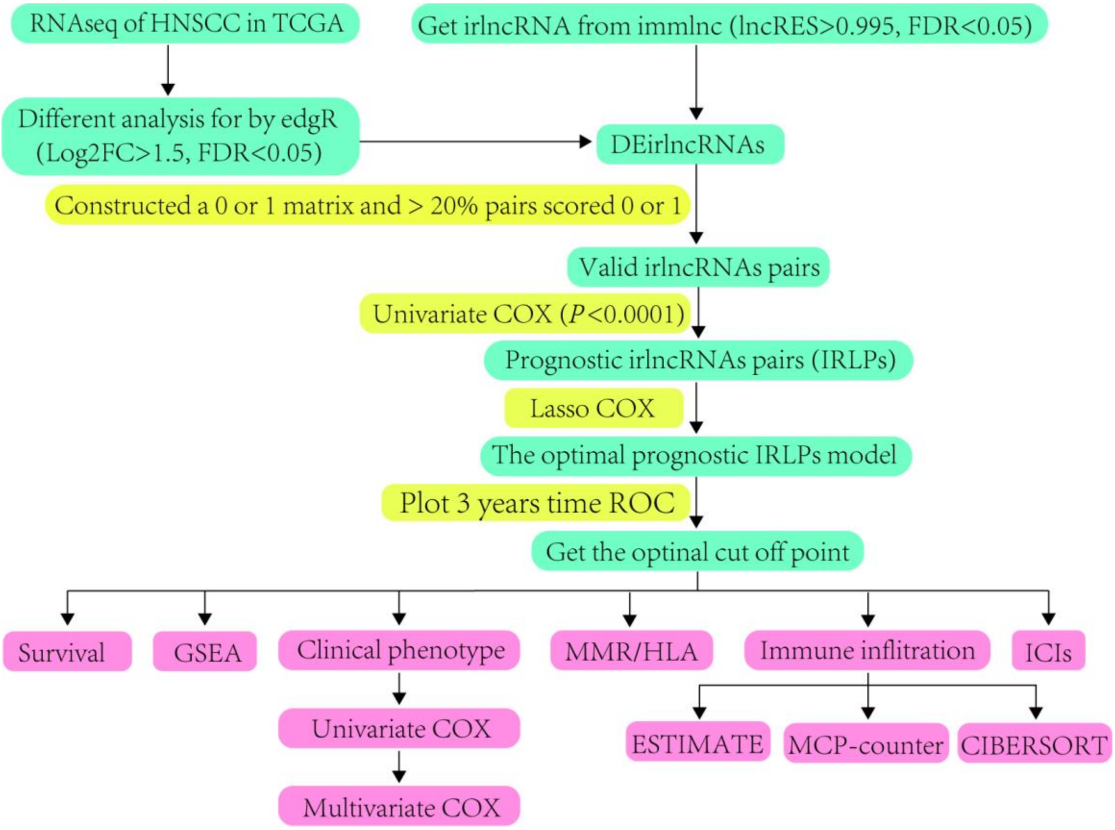

Supplementary Figure 1. Study flowchart showing the process of constructing the 21 IRLPs to predict prognosis of HNSCC.
